# Low-intensity pulsed ultrasound improves symptoms in patients with Buerger disease: a double-blinded, randomized, and placebo-controlled study

**DOI:** 10.1038/s41598-024-64118-0

**Published:** 2024-06-14

**Authors:** Farina Mohamad Yusoff, Masato Kajikawa, Takayuki Yamaji, Shinji Kishimoto, Tatsuya Maruhashi, Ayumu Nakashima, Toshio Tsuji, Yukihito Higashi

**Affiliations:** 1 Department of Regenerative Medicine, Division of Radiation Medical Science, Research Institute for Radiation Biology and Medicine, Hiroshima University, 1-2-3 Kasumi, Minami-Ku, Hiroshima, 734-8551 Japan; 2 Division of Regeneration and Medicine, Medical Center for Translational and Clinical Research, Hiroshima University Hospital, Hiroshima, Japan; 3 Department of Nephrology, Graduate School of Medicine, University of Yamanashi, Yamanashi, Japan; 4 Graduate School of Engineering, Hiroshima University, Hiroshima, Japan

**Keywords:** Randomized controlled trials, Peripheral vascular disease

## Abstract

Here we report the effects of low-intensity pulsed ultrasound (LIPUS) on symptoms in peripheral arterial disease patients with Buerger disease. A double-blinded and randomized study with active and inactive LIPUS was conducted. We assessed symptoms in leg circulation during a 24-week period of LIPUS irradiation in 12 patients with Buerger disease. Twelve patients without LIPUS irradiation served as controls. The pain intensity on visual analog score was significantly decreased after 24-week LIPUS treatment. Skin perfusion pressure was significantly increased in patients who received LIPUS treatment. There was no significant difference in symptoms and perfusion parameters in the control group. No severe adverse effects were observed in any of the patients who underwent LIPUS treatment. LIPUS is noninvasive, safe and effective option for improving symptoms in patients with Buerger disease.

## Introduction

Chronic limb-threatening ischemia (CLTI), or recently described as chronic/critical limb-threatening ischemia, is a complex and severe peripheral artery disease (PAD) that includes atherosclerotic PAD and thromboangiitis obliterans or Buerger disease and treatment options are still being examined^1,2^. New strategies for angiogenesis, such as cell therapy and gene therapy, have been investigated as treatment options for CLTI^2–6^. However, both cell therapy and gene therapy are invasive, and there are current issues of sophisticated handling as well as some safety concerns. Results of such therapies in patients with CLTI are not always satisfactory. Therefore, a novel strategy for therapeutic angiogenesis that is noninvasive and safe and improves symptoms in PAD patients with a wide range of limb ischemia severities is needed.

Low-intensity pulsed ultrasound (LIPUS) irradiation has been used to promote the healing of bone fractures in humans^7–9^. It is postulated that the intitial step of bone fracture healing by LIPUS is angiogenesis, and subsequent neovascularization leads to the migration of osteoblasts to the bone fracture site. Indeed, a few studies have shown that LIPUS induces angiogenesis in experimental murine hind-limb ischemia models^10–12^. However, there is no information on the effects of LIPUS on angiogenesis in humans. We hypothesized that LIPUS irradiation has beneficial effects on limb ischemia by inducing angiogenesis in patients with PAD.

Therefore, in the present study, we evaluated the effects of LIPUS on clinical symptoms in PAD patients with Buerger disease.

## Results

Baseline clinical characteristics of patients in the LIPUS group and patients in the control group are summarized in Table 1.

**Table 1:** Clinical characteristics in the control group and the LIPUS group.

Variables	Control group (n = 12)	LIPUS group (n = 12)
Age, year	45.9 ± 8.7	46.7 ± 8.9
Gender, men/women	11/1	10/2
Body mass index, kg/m2	22.5 ± 3.1	22.7 ± 2.9
Rutherford category, n (%)		
3	2 (17)	2 (17)
4	4 (33)	3 (25)
5	5 (42)	6 (50)
6	1 (8)	1 (8)
Complications, n (%)		
Hypertension	2 (17)	3 (25)
Diabetes mellitus	3 (25)	3 (25)
Dyslipidemia	3 (25)	3 (25)
Pervious myocardial infarction	0 (0)	0 (0)
Pervious stroke	0 (0)	0 (0)
Medication, n (%)		
Anti-platelets	9 (75)	8 (67)
ARBs	2 (17)	1 (8)
Calcium channel blockers	1 (8)	1 (8)
Statins	3 (25)	2 (17)
Sulfonylurea and/or metformin	1 (8)	1 (8)
Smoker, n (%)	3 (25)	3 (25)

**Figure 1:**
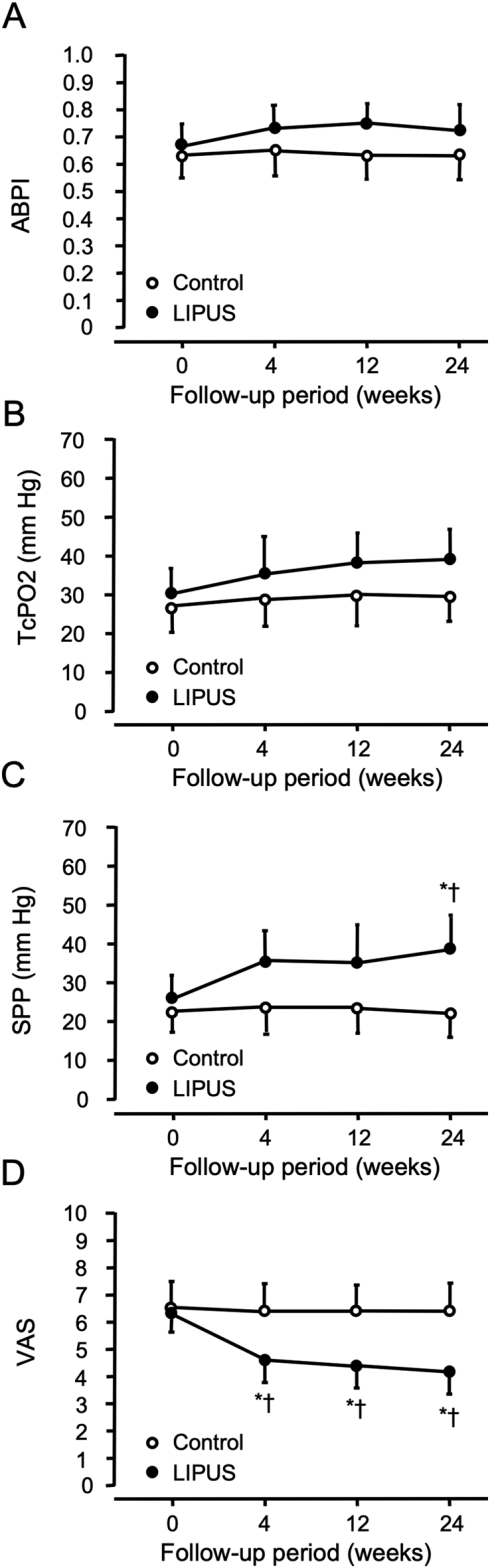
Changes in (**A**) ankle brachial pressure index (ABPI), (**B**) transcutaneous oxygen pressure (TcPO_2_), (**C**) skin perfusion pressure (SPP), and (**D**) visual analog pain score (VAS) in the control (open circles) and low-intensity pulsed ultrasound (LIPUS) groups (closed circles) of patients with Buerger disease. *P < 0.05 vs. 0 weeks in the same group. †*P* < 0.05 vs. Control at the same follow-up period.

Figure 1 shows changes in ABPI, TcPO_2_, SPP, and VAS during LIPUS treatment in the LIPUS group and control group at 0, 4, 12, and 24 weeks and comparison of the parameters between the two groups during the 24-week period of LIPUS treatment. The visual analog score (VAS) score for rest pain was significantly decreased from 6.3 ± 0.4 to 4.7 ± 0.4 (*P* = 0.02) after 4 weeks of LIPUS treatment and it remained low until the end of the 24-week period. LIPUS treatment had a tendency to increase ankle brachial pressure index (ABPI) and transcutaneous oxygen pressure (TcPO2), but not significantly, during the 24-week period. Skin perfusion pressure (SPP) was significantly increased in patients with Buerger disease from 26.3 ± 5.8 to 38.9 ± 8.3 mmHg (*P* = 0.01) after 24 weeks of LIPUS treatment. There was no significant difference between ABPI, TcPO_2_, SPP, or VAS in the control group. There were no significant differences in ABPI and TcPO_2_ in the same follow-up period between the two groups. SPP at the 24 weeks, but not at 0, 4, and 12 weeks, was significantly higher in the LIPUS group than in the control group. VAS at 4, 12, and 24 weeks, but not at 0 weeks, was significantly lower in the LIPUS group than in the control group. Three of the 12 patients had ulcers before entry into this study. Ulcers in the right hallux in two patients were completely cured after LIPUS irradiation (Supplementary Figure S1, A and B).

No severe adverse effects were observed in any of the patients who underwent LIPUS.

## Discussion

Here we report the effects of LIPUS on symptoms in PAD patients with Buerger disease. This clinical trial is the first study showing safety and efficacy of LIPUS for symptoms and perfusion parameters in patients with Buerger disease using a randomized, double-blinded, and placebo-controlled design.

It has been challenging to improve symptoms in patients with severe Buerger by conventional interventional options and they are at risk of amputation^13^. We have shown that cell therapy with bone-marrow mononuclear cell implantation is safe and effective in patients with CLTI, especially in patients with Buerger disease^14^. However, both gene and cell therapies are not always satisfactory in patients with CLTI, and a more effective and simple option is needed. LIPUS may be new therapy for limb ischemia.

In the present study, SPP and symptoms were improved by LIPUS irradiation in patients with PAD. Perfusion parameters, such as ABPI and TcPO_2_, showed a tendency to increase, but not significantly, during the 24-week period of LIPUS irradiation. Some possible mechanisms by which LIPUS improved symptoms in patients with PAD are postulated. It is thought that LIPUS, in vitro and in vivo, induces angiogenesis through increases in angiogenic growth factors and angiogenic cytokines^15–18^. These findings suggest that LIPUS-induced angiogenesis contributes to improvement of perfusion parameters and symptoms in these patients.

Both acute and chronic increases in shear stress patently stimulate the release of nitric oxide (NO) in isolated vessels and cultured cells^19,20^. Ultrasound induces an increase in mechanical shear stress on the surface of endothelial cells^21^. Therefore, ultrasound also may act as a mechanical stimulator of NO release. Indeed, Suchkova et al.^11^ have shown that vasodilatory effects of 40 kHz LIPUS at 0.75 W/cm^2^ are completely abolished by the NO synthesis inhibitor N^G^-nitro-L-arginine-methyl-ester in a rabbit hind-limb ischemia model. Chronic increases in shear stress have been shown to lead to functional and histologic alterations of the vascular endothelium, resulting in enhanced vascular structure and function. LIPUS treatment at an intensity of 1.6–2.0 W/cm^2^ for 6 days enhanced NO and Ca^2+^ release from endothelial cells but did not promote endothelial cell growth^22^. Ultrasound stimulation has been shown to change the cellular morphology and orientation and increase the extracellular matrix secretion from endothelial cells. These findings suggest that LIPUS activates NO synthesis through an increase in shear stress and increase in intracellular Ca^2+^ in endothelial cells, leading to angiogenesis.

Previous studies have shown that LIPUS enhances phosphorylation of ERK1/2 in various types of cells such as osteoblasts and chondrocytes^23,24^, C2C12 cells^25^, human embryonic palatal mesenchyme cells^26^, MC3T3-E1 cells^27^, and murine macrophage cells^28^. LIPUS promotes proliferation via the activation of focal adhesion components, including integrin, its receptors and Src, and the Src/Rho-associated kinase/ERK signaling pathway in human fibroblast cells. It is likely that LIPUS induces phosphorylation of ERK1/2 via focal adhesion components in endothelial cells. Toyama et al.^29^ showed that LIPUS of 100 mW/cm^2^ in intensity for 240 s augmented the proliferation of circulating angiogenic cells derived from human peripheral blood mononuclear cells through enhancement of the Akt/eNOS/NO pathway in vitro.

High-intensity ultrasound induces tissue warming, including warming of vasculatures^21^. Thermal effects may be a dual-edged sword for the vasculature. It has been shown that LIPUS has beneficial effects on the vasculature independent of thermal effects. Indeed, LIPUS of 27 kHz continuous wave at 0.25 W/cm^2^ for 10 min increased NO synthase activity and NO production in human umbilical endothelial cells in a temperature-controlled water bath^30^. Miyamoto et al.^31^ showed that although LIPUS of 1.4 W/cm^2^ and a pulse wave with a duty cycle of 30% for 5 min induced coronary vasodilation in dogs, intracoronary temperature showed no change during exposure to LIPUS for 90 min. In a preliminary study, we found that LIPUS used in the present study slightly increased deep temperature by maximal 0.006 °C in skeletal muscle tissue using a simulation model (Supplemental Figure S2 and videos I and II). In the present study, skin temperature did not change in either the LIPUS-exposed or non-exposed legs (36.1 ± 0.3 to 36.2 ± 0.4 °C and 36.3 ± 0.4 to 36.3 ± 0.4 °C, P = 0.79 and P = 0.88, respectively, n = 12) and skin temperatures were similar in the LIPUS-exposed legs and non-exposed legs during the study. These findings suggest that the thermal effect of LIPUS used this study does not contribute to improvement of symptoms in patients with PAD.

This study has a few limitations. First, the number of subjects in the present study was relatively small. However, improvement of symptoms after LIPUS irradiation were observed in patients with Buerger disease. Second, the LIPUS signal and energy used in the present study were used in previous clinical studies showing efficacy for bone fracture healing^7–9^. In experimental murine hind-limb ischemia models, different pulse waves (0.75 MHz to 3 MHz) and energy outputs of ultrasound (5 mW/cm^2^ to 100 mW/cm^2^) and different irradiation times were used to find conditions effective for angiogenesis^10–12^. An optimal ultrasound treatment method for therapeutic angiogenesis remains unclear. Establishment for optimal ultrasound treatment would enable more specific conclusion concerning the role of ultrasound in angiogenesis in humans to be drawn. Third, although no severe adverse effects were observed after LIPUS irradiation in the present study, further studies are needed to evaluate adverse effects and outcomes, including not only cardiovascular outcomes but also onset of malignancy. A longer follow-up period using a prospective randomized controlled study design is needed. Fourth, in the present study, the mechanisms by which LIPUS improves symptoms in patients with Burger disease remain unclear. Further study is needed to evaluate the mechanisms of LIPUS-induced improvement in symptoms in humans.

In conclusion, LIPUS irradiation is noninvasive and safe and it improves symptoms in patients with Buerger disease. LIPUS may be a new strategy for improving ischemic limb symptoms. It is expected that the use of LIPUS as an option for PAD patients with a wide range of limb ischemia severities. Further studies are needed to evaluate the effects of long-term LIPUS treatment on adverse effects and various events, including not only cardiovascular outcomes but also onset of malignancy, using a prospective randomized controlled study design.

## Methods

### Subjects

**Figure 2:**
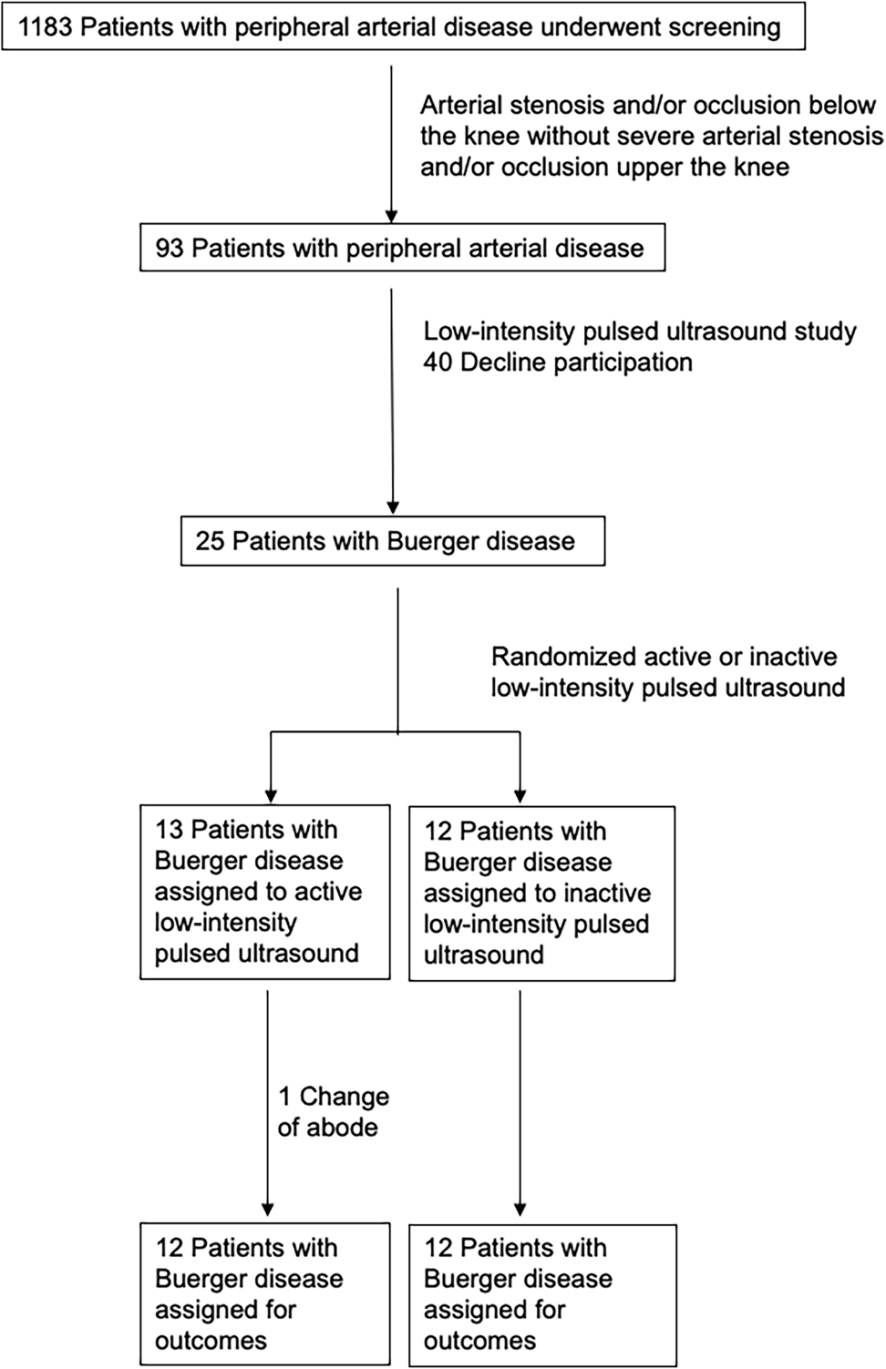
Flow chart of the study design from screening to completion of the trial.

Twenty-five patients PAD with Buerger disease, with Rutherford 3 to 6 from 1183 patients in the Hiroshima University PAD database, were enrolled in this study (Fig. 2). The recruitments were performed after institutional review board approval, registered and updated with Japan Registry of Clinical Trial (jRCTs062200008), and followed up until 31/07/2022 in accordance with the study protocol schedule. Written informed consent was acquired from all subjects. Buerger disease was diagnosed by previous criteria^15^ through physical examinations, symptoms, and angiographic findings of infrapopliteal arterial occlusive disease, either upper limb involvement or phlebitis migrans, along with history of smoking habit, onset before the age of 50 years and absence of atherosclerotic risk factors other than smoking. To rule out other vascultitis and hypercoagulable states, rheumatoid factor, lupus anticoagulants, and serologic investigations were evaluated. Limb ischemia diagnosis was confirmed by angiography.

### Study protocol

This study was a double-blinded and randomized study with active and inactive LIPUS. To perform the inactive LIPUS study, we prepared a dummy LIPUS device. The dummy LIPUS devices have been constructed to appear the same in appearance and sounds as the LIPUS device and a dummy indicator lights up in operation. Both the dummy and true devices do not have vibrations. Twenty-five patients with Buerger disease were randomly divided into an active LIPUS group and an inactive LIPUS group. Patients continued to receive conventional therapy during LIPUS treatment. In addition, there were no changes or additions of medications during the observation period after LIPUS treatment. Other than main researchers of this study, the observers and patients are blinded to the form of examination. The study protocol was approved by the Ethics Committee of Hiroshima University Graduate School of Medicine and research have been performed in accordance with the Declaration of Helsinki. Written informed consent for participation in the study was obtained from all subjects.

### Randomization and masking

Limb ischemia diagnosis was confirmed by angiography. Digital subtraction angiography (DSA) results that demonstrated angiographic findings of infrapopliteal arterial occlusive disease and confirmed diagnosis of Buerger disease are the inclusion criteria for study subject recruitment. These eligible patients were randomly assigned to receive treatment with either active LIPUS equipment (Nippon Sigmax Co., Ltd, Tokyo, Japan) or dummy LIPUS equipment (ratio 1:1). The random code list was electrically generated by an independent trial statistician where the clinical center was assigned as block by a third-party enrollment center.

### Endpoints

The primary endpoint of the trial is change in rest pain intensity on a VAS from baseline to 24 weeks. The secondary endpoints are change in rest pain intensity on the VAS from baseline to 12 weeks, changes in TcPO_2_, SPP and ABPI from baseline to 12 and 24 weeks, and change in symptoms and perfusion parameters related to subjects in the study period of 24 weeks.

### LIPUS treatment

We made a device for LIPUS therapy (Supplemental Figure S3). Detailed specifications of the LIPUS device are presented in Supplemental Figure S4. The ultrasound irradiation was applied over the skin at the calf region of the ischemic limb at home 20 min every day for 24 weeks. The gap between the transducer and skin was filled with ultrasonic gel. LIPUS device with individual transducer (Nippon Sigmax Co., Ltd, Tokyo, Japan) generating a pulsed 2.0-MHz signal with a 200-μ second pulse burst and 1-kHz repetition rate at the intensity of 30 mW/cm^2^ and with a duty cycle of 20% of pulse wave. In the present study, 8 transducers were attached to the skin over the gastrocnemius of each ischemic leg (Supplemental Figure S3).

### Digital subtraction angiography (DSA)

All of the subjects underwent intra-arterial DSA in the course of examination for diagnosis of PAD. Briefly, conventional DSA was performed with a 4-F pigtail catheter (TERUMO, Tokyo, Japan) by using DSA units (Siemens-Asahi Medical, Tokyo, Japan). The tip of the catheter was positioned at the common iliac arteries and images that included the thighs, knees and crural regions were captured. At each station, 20 mL of iopromidum was injected at a rate of 10 mL/sec.

### Duplex ultrasonography

Lower extremity arterial duplex scanning was performed in all patients. Briefly, all of the duplex ultrasonography scans were performed by ultra-sonographic specialists who were aware of the angiographic findings of DSA. The ultrasound unit Aloka-α7 (Aloka, Tokyo, Japan) equipped with a linear, phased-array high-frequency (13-MHz) transducer was used for scanning peripheral arteries. Arterial duplex scanning was started at the femoral artery in the recumbent position. The transducer was placed at the inguinal ligament and then moved distally to the knee, following the course of the superficial arteries. Then the transducer was repositioned below the knee following the course of anterior tibia arteries and moved distally to the ankle. The popliteal arteries were examined in the prone position. A diagnosis of occlusion of a popliteal artery was made on the basis of lack of flow signal at the usual place of original arteries.

### Measurement of VAS, TcPO_2_, ABPI, SPP

#### VAS

Rest pain intensity was assessed by a self-administered VAS from baseline to 24 weeks as previously described^16,32^. Briefly, observers were asked to place a marker on the VAS line at a point that represents the pain intensity of respondents. The respondents completed the VAS line presentation by themselves within 1 min. The VAS score was a horizontal line of 10 cm in length, with 0 cm corresponding to no pain and 10 cm corresponding to most severe pain. The observers were blinded to the manner of examinations.

### Perfusion parameters, TcPO_2_, SPP, and ABPI

All of the examinations were performed in a dimmed-lighted, air-conditioned and quiet room in the morning after overnight fasting (constant temperature of 22–25 °C). The subjects remained in a supine position throughout the study. Perfusion parameters were measured after a 15-min rest period. TcPO_2_ was measured by an electrochemical transducer (TCM 400, Radiometer K.K., Tokyo, Japan) as previously described^33^. Briefly, skin at the measured site was shaved and cleaned by alcohol to ensure optimal TcPO_2_ electrode contact for skin oxygen diffusion. Then the TcPO_2_ electrodes stabilized at 43 °C were placed on the skin at the measured site. TcPO_2_ values were recorded after a 20-min stabilization period for the electrodes. SPP was measured by a SensiLase PAD3000 flowmeter (Kaneka Medix Co., Osaka, Japan) as previously described^34^. Briefly, a laser Doppler probe positioned within the blood pressure cuff was wrapped around the subject’s foot. ABPI was measured by a new device (Form PWV/ABI; Omron Colin, Co., Tokyo, Japan) as previously described^35^. Briefly, the subjects were kept in the supine position for at least 5 min and cuffs were wrapped around both brachia and ankles. An oscillometric method was used to measure systolic blood pressure in the bilateral brachial and posterior tibial arteries. The ABPI value was calculated by dividing the highest pressure in the posterior tibial arteries on the right and left sides by the highest brachial pressure on either side. All procedures were performed in accordance with the instructions from manufacturers. The observers were blind to the manner of examinations.

### Statistical analysis

Results are presented as the mean ± SD. All reported *P* values were two-tailed. Values of *P* < 0.05 were considered significant. Continuous variables were compared by using analysis of variance for multiple groups and the t-test between two groups. Categorical variables were compared by the Fisher’s exact test. Comparisons of variables before and after LIPUS were performed with adjusted means by analysis of covariance with the use of baseline data as covariates. Comparisons of time course curves of ABPI, TcPO_2_, SPP, and VAS with 10 levels after LIPUS were performed using two-way ANOVA for repeated measures on one factor followed by Bonferroni correction for multiple-paired comparisons. Missing ABPI, TcPO_2_, SPP, and VAS values were imputed using last observation carried forward method. Data were processed using the software package Statview (SAS Institute, Cary, NC, USA) or Super ANOVA (Abacus Concepts, Berkeley, CA, USA).

### Supplementary Information

Supplementary Information 1.

Supplementary Information 2.

Supplementary Video 1.

Supplementary Video 2.

## Data Availability

The data that support the findings of this study are not openly available due to reasons of sensitivity and are available from the corresponding author upon reasonable request. Data are located in controlled access data storage at Hiroshima University.
