# Supplementary material for: Low-intensity pulsed ultrasound improves symptoms in patients with Buerger disease: a double-blinded, randomized, and placebo-controlled study
Source: Sci Rep. 2024 Jun 14;14:13704. doi: 10.1038/s41598-024-64118-0 (PMC11176328; doi:10.1038/s41598-024-64118-0)
Supplement: Supplementary file 1 — Supplementary Information 1. [file 41598_2024_64118_MOESM1_ESM.docx]

**Legend for video files**

**Supplementary Figure S2 (Video I and II)**

(B) Visible pulse of LIPUS irradiation in the lower leg (see video I).

(C) Visible changes in temperature in muscle during LIPUS irradiation (see video II).
