# Supplementary material for: Low-intensity pulsed ultrasound improves symptoms in patients with Buerger disease: a double-blinded, randomized, and placebo-controlled study
Source: Sci Rep. 2024 Jun 14;14:13704. doi: 10.1038/s41598-024-64118-0 (PMC11176328; doi:10.1038/s41598-024-64118-0)
Supplement: Supplementary file 2 — Supplementary Information 2. [file 41598_2024_64118_MOESM2_ESM.docx]

**Supplementary Appendix**

**Low-intensity Pulsed Ultrasound Improves Symptoms in Patients with Buerger Disease: A Double-blinded, Randomized, and Placebo-controlled Study**

Short title: LIPUS and Symptoms in Buerger Disease

Farina Mohamad Yusoff, MBBS, PhD,^1^ Masato Kajikawa, MD, PhD,^2^ Takayuki Yamaji, MD, PhD,^1^ Shinji Kishimoto, MD, PhD,^1^ Tatsuya Maruhashi, MD, PhD,^1^ Ayumu Nakashima, MD, PhD,^3^ Toshio Tsuji, PhD,^4^

Yukihito Higashi, MD, PhD^1,2^

^1^Department of Regenerative Medicine, Division of Radiation Medical Science, Research Institute for Radiation Biology and Medicine, Hiroshima University, Hiroshima, Japan

^2^Division of Regeneration and Medicine, Medical Center for Translational and Clinical Research, Hiroshima University Hospital, Hiroshima, Japan

^3^ Department of Nephrology, Graduate School of Medicine, University of Yamanashi, Yamanashi, Japan

^4^Graduate School of Engineering, Hiroshima University, Hiroshima, Japan

Address for correspondence:

Yukihito Higashi, MD, PhD, FAHA

Department of Regenerative Medicine, Division of Radiation Medical Science, Research Institute for Radiation Biology and Medicine, Hiroshima University

1-2-3 Kasumi, Minami-ku, Hiroshima 734-8551, Japan

Phone: +81-82-257-5831 Fax: +81-82-257-5831

E-mail: [yhigashi@hiroshima-u.ac.jp](mailto:yhigashi@hiroshima-u.ac.jp)

**Supplementary Figure S1**

**
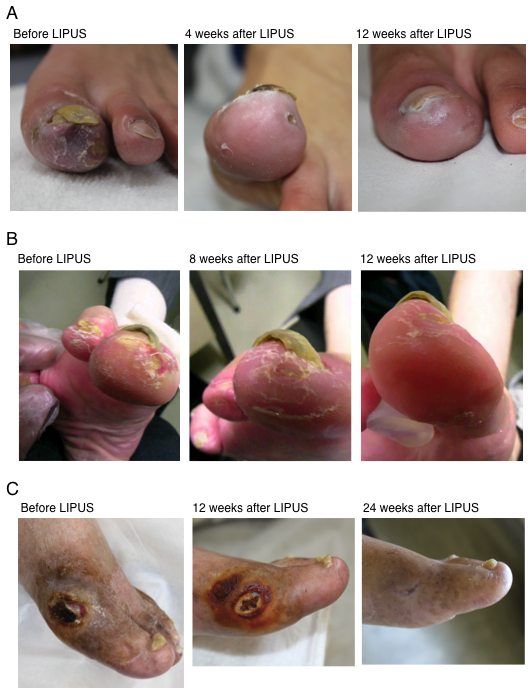
**

**Supplementary Figure S1 Legend.**

Representative data on low-intensity pulsed ultrasound (LIPUS)-induced cure of ulcers in the right hallux of a patient with Buerger disease at the beginning of treatment (before) and after 4 and 12 weeks of treatment.

**Supplementary Figure S2 (Video I and II)**


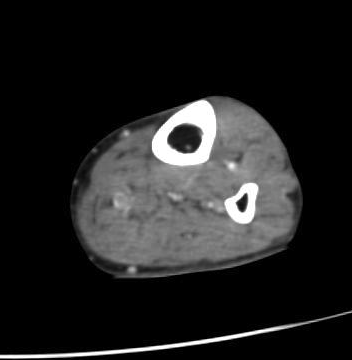


A

Bone

Muscle

Artery

Vein

Air

Tissue

Fat

LIPUS


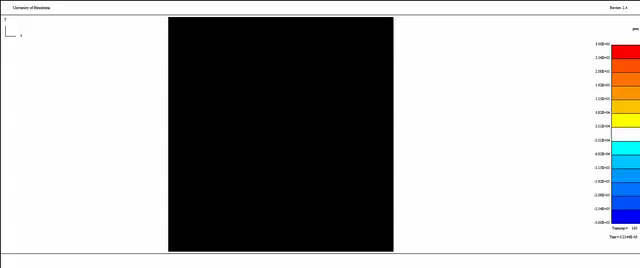
B **Video I**


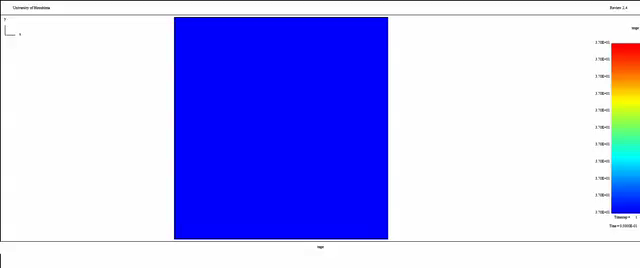
C **Video II**

D

Temperature (℃)

5

10

25

37.000

37.001

37.002

37.003

37.004

37.005

37.006

37.007

37.008

37.009

Time (second)

36.999

0

15

20

1200

**Supplementary Figure S2 Legend.**

(A) Simulation model for low-intensity pulsed ultrasound (LIPUS) irradiation (2.0 MHz signal with 200 μ seconds pulse burst, 1 kHz repeat rate at the intensity of 30 mW/cm^2^, and duty cycle 20%) in the lower leg. (B) Visible pulse of LIPUS irradiation in the lower leg (see video I). (C) Visible changes in temperature in muscle during LIPUS irradiation (see video II). (D) Changes in temperature in muscle during LIPUS irradiation.

**Supplementary Figure S3**

**
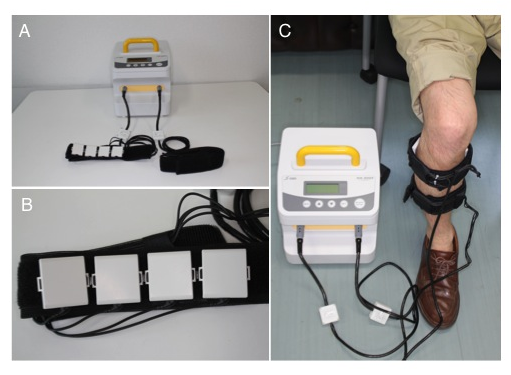
**

**Supplementary Figure S3 Legend.**

Content of specification for therapeutic angiogenesis device using a low-intensity pulsed ultrasound (LIPUS) system.

**Supplemental Figure S4**

**Composition**

Main body

AC power cord

Transducer set: 2 sets (each set consists of 4 transducer elements or cells)

Ultrasonic gel

Fixing band: 2

**Specifications**

Main body size: width, 240 mm; depth, 245 mm; height, 263 mm

Weight: 5.5 kg

Power supply: 100 VAC~240VAC±10%

Electricity consumption: ≤ 150 VA

**Ultrasound output specifications**

Ultrasound transducer element size: width, 34.68 mm; depth, 38.68 mm; height, 7.84 mm

Ultrasound transducer element weight: 17.0 g

Ultrasound transducer element number: 8 elements (each transducer consists of 4 elements)

Ultrasound frequency: 2 MHz±10%

Ultrasound output power (ISATA): 30 mW/cm^2^

Beam non-uniformity ratio: 5±2

Pulse duration: 200 μs±5%

Pulse repetition frequency: 1 kHz±5%

Pulse duty: 20%±5%

Output duration: 20 minutes

Main body


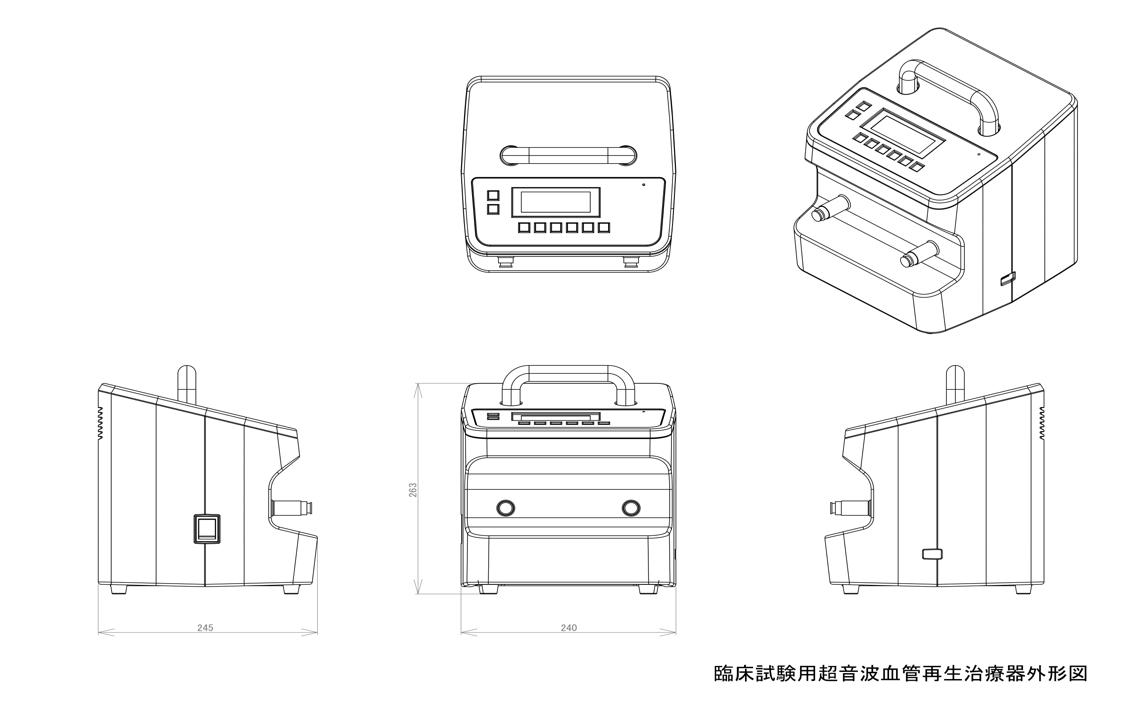


Ultrasound transducer element


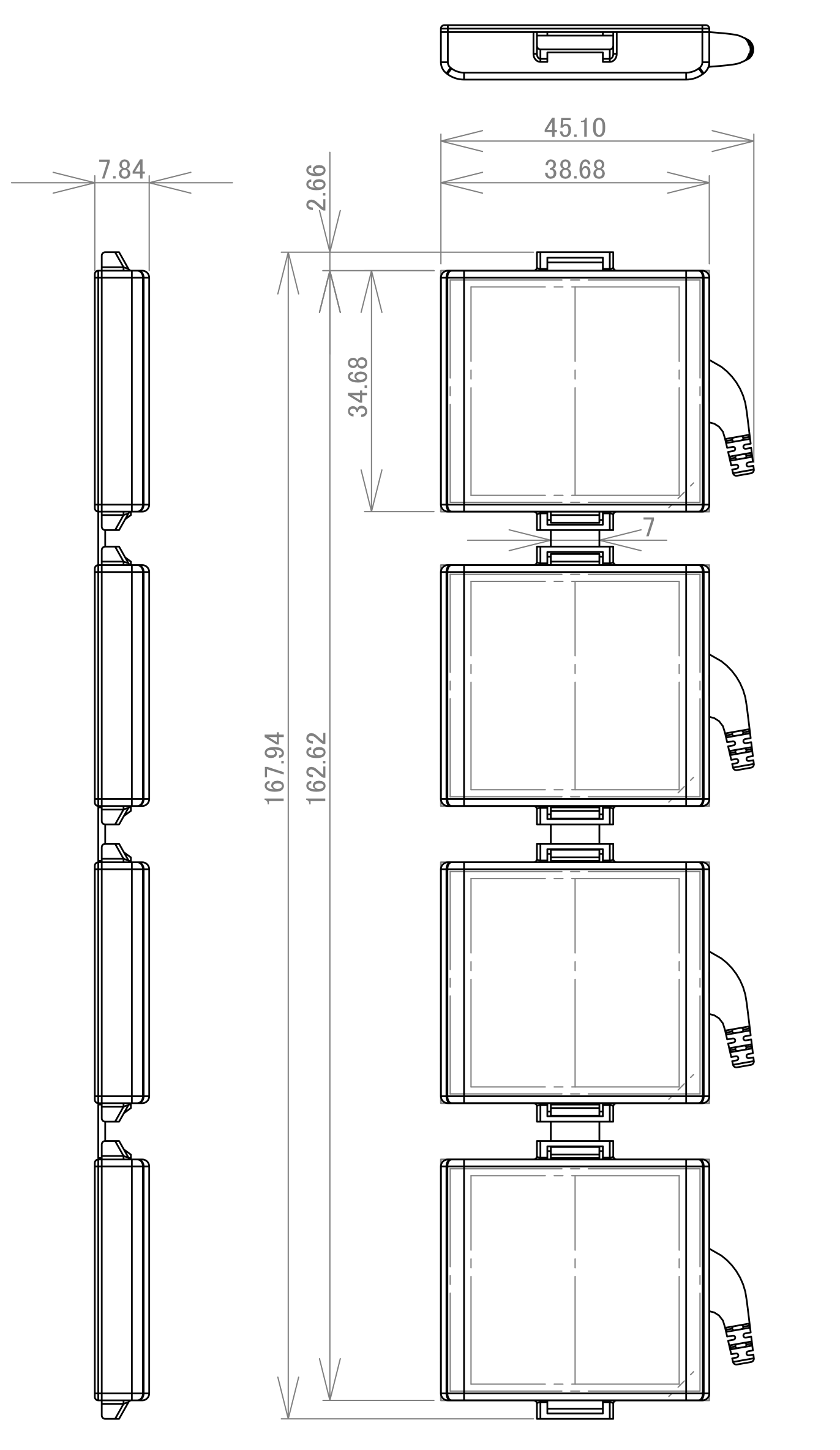


Ultrasound control mode: burst wave time-sharing system

Each channel or group consists of 4 transducer elements. Within a channel each transducer element is driven one by one with the power generator so that convoluation of emitted ultrasound is unlikely. Ultrasound output order was as follows.

**Supplemental Figure S4 legend.**

Content of specification for therapeutic angiogenesis device using a low-intensity pulsed ultrasound (LIPUS) system.
